# Experimental Research on the Influence of Different Curved Rigid Boundaries on Electric Spark Bubbles

**DOI:** 10.3390/ma13183941

**Published:** 2020-09-06

**Authors:** Chunlong Ma, Dongyan Shi, Yingyu Chen, Xiongwei Cui, Mengnan Wang

**Affiliations:** 1 College of Mechanical and Electrical Engineering, Harbin Engineering University, Harbin 150001, China; wangmengnan@hrbeu.edu.cn; 2 Department of Automotive, Harbin Vocational & Technical College, Harbin 150000, China; 3 College of Shipbuilding Engineering, Harbin Engineering University, Harbin 150001, China; chenyingyuhrbeu@gmail.com (Y.C.); cuixiongwei@yahoo.com (X.C.)

**Keywords:** *ζ*, *γ*, load characteristics, bubble shrink shape, jet, bubble’s first pulsation period

## Abstract

It is well known that the bubble dynamics and load characteristics of cavitation bubbles depend to a great extent on their proximity to the boundary. The purpose of this study is to explore the relationship between the boundary curvature and bubble dynamics, as well as the load characteristics, and summarize the relevant change laws. This study takes three hemispheres of different curvatures and one flat board as its main research boundaries. The hemisphere was chosen as the curved surface boundary because the hemisphere represents the simplest type of curved surface boundary. This method allowed us to easily observe the experimental results and summarize the change laws of bubble dynamics and load characteristics. A high voltage electricity of 400 V was used to produce stable and repeatable electric spark bubbles in this experiment. Since the pulsation time of the bubbles is very short, we used a high-speed camera to acquire the necessary photographs. We also used a Hopkinson bar (HPB) to measure the bubble collapse load. Suppose that the dimensionless parameter of curvature is ζ and the dimensionless parameter of the explosion distance is γ. By summarizing the 44 groups of the experimental results under different combinations of ζ and γ, we found that the cavitation bubble dynamics and loading characteristics are affected by ζ. With an increase of ζ, the shockwave load and bubble collapse load will decrease. In addition, in terms of load characteristics, this study further verified the change trend of the shockwave load and bubble collapse load with γ. For the bubble shrink shape, this paper illustrates the relationship between the bubble’s shrink shape and its shrinkage speed. Four typical bubble shrink shapes are summarized. The effects of different ζ and γ values on the jet are preliminarily explored using the experimental results, and, by considering the experimental results, the developmental trends of the time of the bubble’s first pulsation period are discussed.

## 1. Introduction

Bubbles’ pulsation characteristics near object surfaces are widely studied in many fields, such as underwater explosions [1,2], damage to propellers by cavitation bubbles [3,4], ultrasonic cleaning [5,6], medical treatment [7], etc. Many previous studies proved that boundary conditions (such as fixed boundaries [8,9,10], free surfaces [11,12,13], elastic boundaries [13], and motion boundaries [14]) greatly affect the bubbles’ pulsation characteristics.

A fixed boundary is the most common boundary condition. As early as the 1960s, scholars [15] had determined the morphological evolutionary characteristics of bubbles nearby fixed boundaries. Under the influence of a fixed boundary, a bubble will form a jet directed at the fixed boundary and a local high pressure region during the contraction phase; the formed jet load will then directly act on the fixed boundary. In the study of Tomita et al. [16], the characteristics of bubble dynamics under the influence of a curved surface boundary are given from theoretical and experimental perspectives. Their study also showed that “If a boundary is slightly concave, the most pronounced migration occurs at the first bubble collapse. The velocity of a liquid jet impacting on the far side of the bubble surface tends to increase with increasing boundary curvature. In the case of a convex boundary, the jet velocity is larger than that generated in the flat boundary case [16]”. For a spherical boundary, Li Shuai et al. [17,18] established a bubble-sphere full coupling numerical calculation model based on the boundary element method and carried out a related experiment to verify the shape and motion of the bubble. This research showed that the established numerical method can accurately represent the morphological evolutionary characteristics of bubbles near the sphere boundary and also provided many detailed morphological characteristics. Ma Chunlong et al. [19] gave the explosion distance parameter that affects the bubble load characteristics near the hemisphere boundary using the Hopkinson bar (HPB). However, few scholars have provided the load characteristics of bubbles near a curved surface boundary from an experimental research perspective, as most only provide some flow field characteristics in numerical terms. Therefore, based on our previous research, this paper adds another control parameter, *ζ*, for supplementary discussion.

Due to their high repeatability, low cost, short cycle, etc., electric spark bubbles have become a stable bubble generation source and are widely used in bubble characteristics research [20,21]. However, when studying the electric spark bubble load and using common piezoelectric sensors to measure wall pressure at short distances, strong electrical interference often occurs, making the measurement results unusable. When measuring at close range, other problems can occur, such as exceeding the sensor measuring range or the sensor being easily damaged by the current. Therefore, there are few studies on the measurement of electric spark bubbles with piezoelectric sensors [22,23]. According to the requirements of electric spark bubbles and close-range measurements, this paper proposes to measure the underwater bubble load using HPB measurement technology. In the research of S.E. Rigby [24], using a large number of air explosions and soil explosion experiments, the HPB was able to obtain the “pressure–time” curve of the explosive shockwave load. Cui Xiongwei [25] used HPB to study underwater explosions. Subsequent research has proven that the measured values of the measurement system are stable and have clear advantages in the measurement of short-range explosion shockwave loads and high-voltage electric spark bubble loads [26,27].

Our previous paper [19] studied the design of a new wall pressure load measurement system based on the work of Yao and Cui [26,27]. By changing the shape of the target plate, the morphology and load characteristics of bubbles produced by near-field underwater explosions were studied and analyzed under curved surface boundary conditions. In the study in our previous paper, we investigated the bubble loading characteristics under the same boundary conditions and at different explosion distances and provided a detailed discussion and analysis of the effectiveness and scope of the test system. Based on this, this paper mainly studies the bubble dynamics and load characteristics under the influence of two control parameters, *ζ* and *γ*, and provides a reference for further studying the quantitative relationships of bubble loads near curved surface boundaries.

## 2. Experimental Setup

### 2.1. HPB

Since aluminum, steel, and copper are common materials that have been deeply studied, we selected them as HPB materials. The physical properties of aluminum, steel, and copper are shown in Table 1. The elastic modulus of aluminum is smaller than that of steel and copper. When aluminum is subjected to pressure, a larger strain response will occur, allowing the weaker stress wave signal propagating within the aluminum to be more easily detected by a measurement device. Since an underwater electric spark explosion is an underwater explosion with a low intensity, it generates a small pressure load. Therefore, to accurately measure the pressure load, the HPB material used in this experiment was an aluminum alloy.

**Figure 1:**
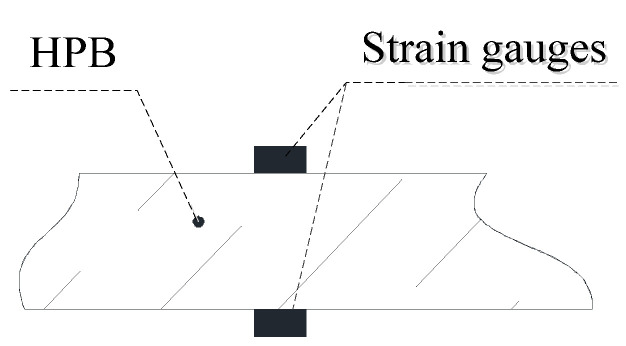
Pair of opposed dual-gauges.

In the actual measurements, a pressure load acts on the HPB’s measurement end. The pressure load will cause the HPB to vibrate. At this time, the bending wave generated by the vibration in the HPB will affect the experimental results. This experiment applies double strain gauges to eliminate these vibrations. Figure 1 shows the location of the opposing dual strain gauges. When the HPB is bent upward or downward, the upper and lower strain gauges will produce opposite and equal resistance changes. These changes can eliminate non-axial strain signals in the HPB and only retain the axial strain signals. Therefore, although two strain gauges were pasted onto the HPB in this experiment, there was only one measuring point.

**Figure 2:**
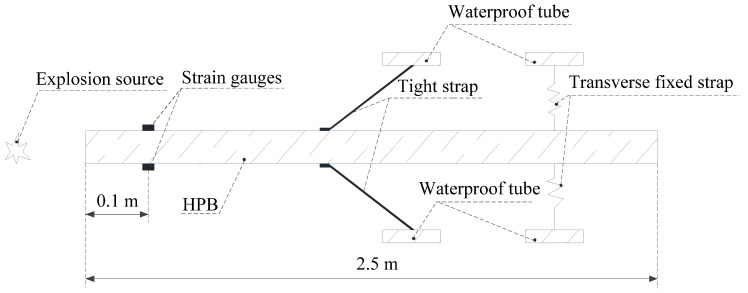
The fixation method for the Hopkinson bar (HPB).

An underwater explosion will generate a shockwave load and a bubble collapse load. If the HPB is not fixed, the HPB will be displaced after being subjected to a pressure load. To avoid this, the HPB should be fixed. Since a rigidly fixed HPB will affect the stress wave measurements, the longitudinal and lateral portions of the HPB are elastically fixed. The research of Xiongwei Cui et al. found that the elastic fixing between the HPB and the protective cylinder through the strong band is very good, so this study used the same method of fixation. The longitudinal and horizontal elastic fixation is shown in Figure 2. 

### 2.2. HPB Measuring System

**Figure 3:**
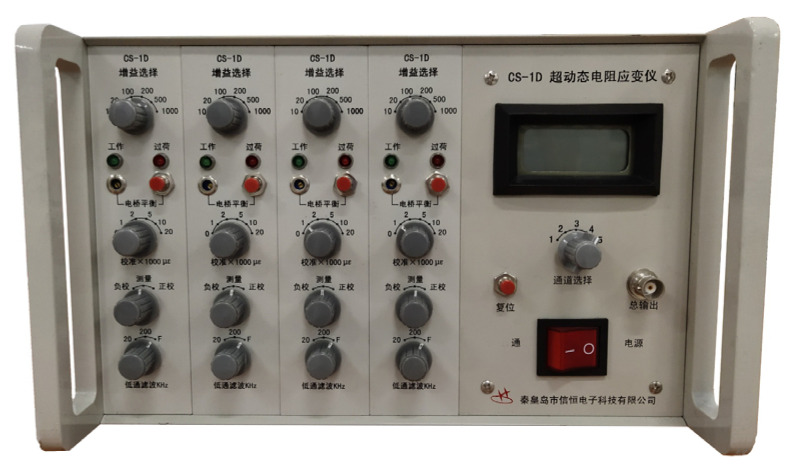
Dynamic resistance strainometer.

Similar to a measurement system using pressure sensors, the measurement system on the HPB is composed of sensors, sensor signal conditioners, and data acquisition systems. The semiconductor strain gauge converts the stress wave signal on the HPB into a resistance signal. The instrument used to convert resistance signals into voltage is a strainometer. Thus, the strainometer acts as a sensor signal conditioner in the strain measurement system. The pressure load generated by the near-field underwater explosion has the characteristics of a short duration, high frequency, and ultra-high frequency. Therefore, the strainometer used in this experiment needed to have a higher frequency response, i.e., a dynamic or super dynamic strainometer was required. The strainometer used in this study was a CS dynamic resistance strainometer produced by Xinheng Electronic Technology Co., Ltd. Xinheng Electronic Technology Co., Ltd is located in Qinhuangdao, China. The maximum frequency response of this strainometer is 1 MHz, which can meet the necessary pressure measurement requirements, as shown in Figure 3.

The pressure load generated by the underwater explosion will be transformed into a stress wave propagating in the HPB after being received by the HPB’s measurement end. The stress wave will propagate from the measurement end along the HPB to the other end, after which, part of the stress wave will be transmitted out, and the other part of the stress wave will be reflected back to the measurement end along the HPB. Each time a stress wave passes through a strain gauge, the strainometer will record the signal and convert it into a pressure load. In other words, the signal generated by the stress wave that first passes through the strain gauge is an accurate signal. When the stress wave reaches the end of the HPB and is reflected to the strain gauge, the signal generated is an inaccurate signal, i.e., the signal generated by the incident wave and the reflected wave superimpose each other. In other words, for an HPB with a fixed length, the accurate pressure load measurement time needs to satisfy the following equation:

(1) $$T\;<\;T_{HPB}\;=\;\left(2\times L_{HPB}-d_{s}\right)⁄C_{V}$$

where *T* is the pulse width of the pressure load (i.e., the measurement time of the pressure load), and $T_{HPB}$ is the pulse width of the mixed wave through which the reflected wave first passes on the strain gauge and then mixes with the incident wave. $L_{HPB}$ is the length of the HPB, and $d_{s}$ is the distance from the HPB measurement end to the strain gauge. The length of the HPB is 2.5 m, and the distance from the place where the strain gauge is attached to the measurement end of the HPB is 0.1 m, as shown in Figure 2. $C_{V}$ is the propagation speed of the stress wave in HPB, $C_{V}\;=\;5.1\;\mathrm{km}/s$. We can use Equation (1) to calculate $T_{HPB}$ = 0.96 ms. Therefore, if *T* is less than 0.96 ms, then the measured value is considered valid.

### 2.3. Rigid Boundary Condition Setting

The rigid boundary used in the experiment consisted of a round flat plate with a diameter of 250 mm, a hemisphere with a diameter of 50 mm, a hemisphere with a diameter of 100 mm, and a hemisphere with a diameter of 150 mm, all of which were made from aluminum alloy. The flat plates were 10 mm thick, with a circular through-hole placed in the center and on the hemisphere. The circular through-hole was used to place the HPB for pressure measurements.

**Figure 4:**
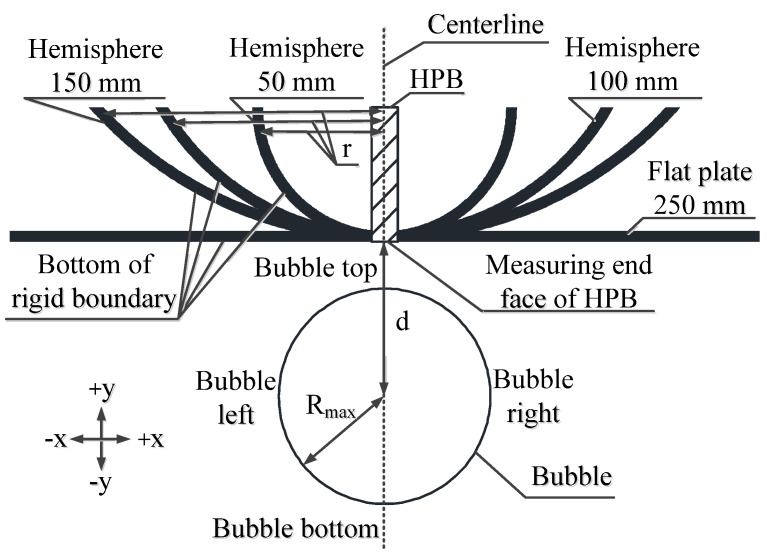
The cross-sectional geometric details of the rigid boundary and description of the directions.

Figure 4 shows the geometric details of the boundary cross section. In Figure 4, *d* represents the explosion distance, that is, the distance between the HPB measurement end-face and the detonation point of the underwater explosion, and *r* represents the radius of the hemisphere boundary. The *r* value of the flat plate is considered to be infinite (∞). The measurement end-face of HPB and the bottom of the rigid boundary are collectively referred to as the ‘bottom of the boundary’ in the following sections.

**Figure 5:**
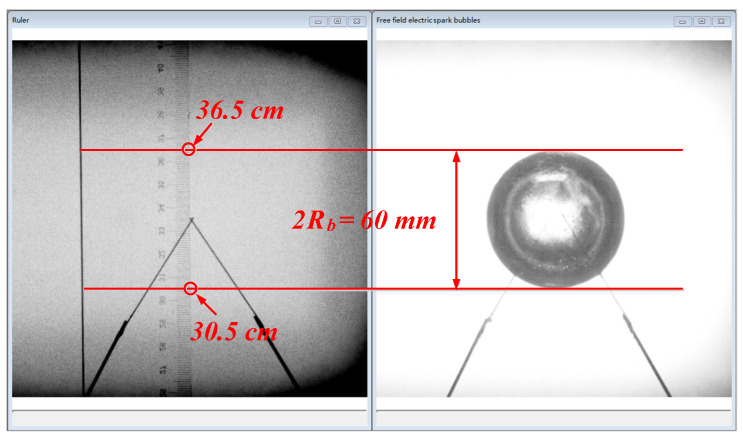
Schematic diagram of the method for measuring the bubble diameter.

Due to the limitations in the principle of stress wave measurements, the measurement end-face of the HPB must be processed into a plane. Therefore, a very small platform with an area of 1.9625 × 10^−5^ m^2^ was produced in the vertical direction. The area of the platform accounted for 0.5%, 0.125%, and 0.055% of the area of the three curved surfaces boundary, respectively. In this way, it can be approximately assumed that the platform is part of a curved surface boundary. The rule in Figure 5 functioned as the measurement standard for the images taken by the high-speed cameras and was mainly used to measure the explosion distance values and bubble sizes.

The experiment is fully 3-D. Another reason for choosing the hemispheres and round flat plates as the boundaries is that they are symmetrical regardless of the viewing angle. Therefore, the bubbles generated at the lower end of the boundary center are also prone to axisymmetric changes. Such bubbles simplify observing the laws of these changes.

### 2.4. Experimental Device

**Figure 6:**
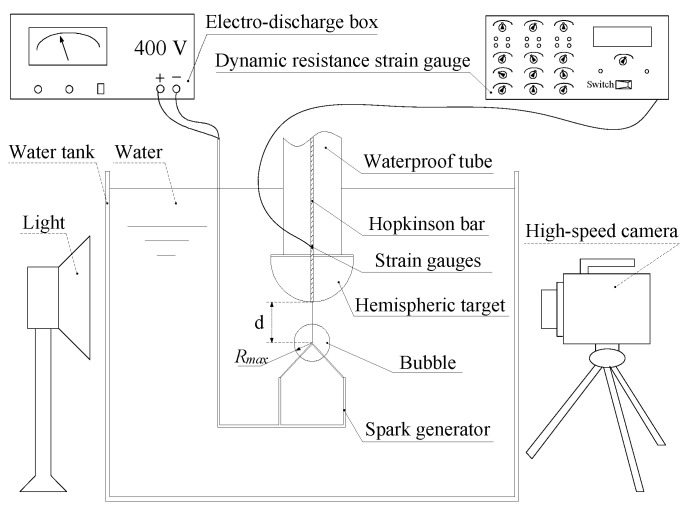
Schematic diagram of the experimental arrangement.

Figure 6 shows the experimental device used for electric spark bubble generation, bubble pulsation process shooting, and the corresponding load measurements near the rigid boundary. Cui et al. [25] used a high-voltage electric discharge to generate bubbles. Ma et al. [19] improved upon the equipment of Cui et al. by adding a replaceable target to realize bubble experiments under different curved surface boundaries. A voltage of 400 V discharged by the capacitor was transmitted to a thin copper tube through a wire, and bubbles were generated at the intersection of the two thin copper tubes. In this experiment, a Phantom VEO-710S high-speed camera and Cannon 16–35 mm optical lens were used to shoot the bubble pulsation process. Phantom VEO-710S high-speed camera is produced by AMETEK in USA.

The highest resolution of this type of high-speed camera is 1024 × 768. With this resolution setting, the shooting speed can reach 7400 frames per second. When the resolution is 512 × 512, the shooting rate can reach 20,000 frames per second. The shooting speed of the Phantom VEO-710S is 30,000 frames/s, and its exposure time is 41–42 μs. Therefore, the Phantom VEO-710S can take 140–144 photos during the bubble’s first pulsation period. Thus, the measurement point of the bubble’s first pulsation period was about 140–144, and the maximum error of the time measurement between each frame of photos was 43 μs. Compared to the bubble’s first pulsation period (about 5–8 ms), this error was sufficiently small. The light source used in the experiment was a 2 kW LED lamp. The experimental water tank glass (which was close to the LED lamp) was frosted glass. As shown in Figure 6, the experimental water tank used in the experiment was a cube tank with a side length of 0.6 m.

The bubble was generated in the center of the water tank, and the size of the water tank was much larger than the bubble’s radius. Therefore, we hypothesized that apart from the rigid boundary and cross electrode, no other boundary would affect the bubble pulsation. The electrode in the experiment used a copper tube with a diameter of 0.5 mm, so its boundary effect was also ignored. The Cannon 16–35 mm optical lens focused on the intersection of electrodes to facilitate the entire process of shooting the bubble pulsation.

### 2.5. Dimensionless Parameter and Bubble Diameter

To discuss the experimental results, we will use the maximum bubble radius ($R_{max}$) to dimensionlessly process the boundary curvature and explosion distance. The explosion distance (γ) and boundary curvature (ζ) are defined as $\gamma =d/R_{max}$ and $\zeta =R_{max}/r$, respectively.

Due to the boundary effect, the bubbles generated near the boundary will deform during the pulsation process. Therefore, the maximum radius of the bubble cannot be accurately measured. There is no boundary effect in the free field, so the maximum radius of the bubble in this state is used as the standard. Assume that $R_{max}$ is the average value of the bubble’s maximum radius generated by a 400 V discharge in the free field. There is a problem in this case. Although the discharge voltage of the electric spark bubbles is stable and repeatable, the vaporization randomness of the copper tube electrode will also affect the bubble size to a certain extent. According to the experimental results, the maximum bubble radius in the free field has a very limited variation range. Therefore, the influence of the vaporization of the copper tube electrode on the bubble radius was ignored here.

**Figure 7:**
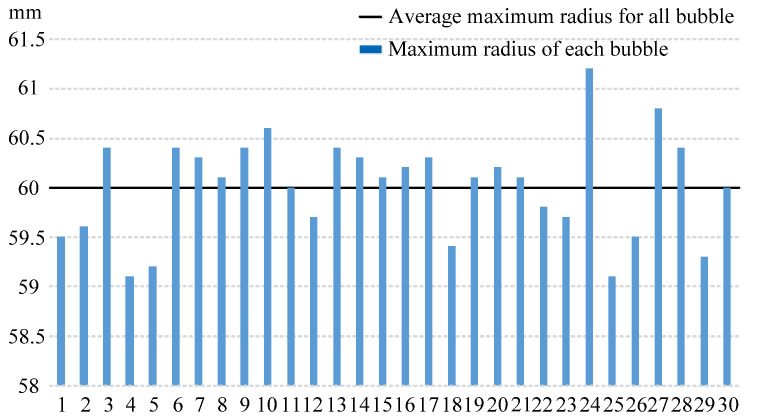
The maximum radius of the bubble generated in the free field through a 400 V discharge. The solid line is the average value of the maximum radius of the bubble.

To obtain the average value of the maximum bubble radius, we conducted 30 sets of bubble experiments with a 400 V voltage in the free field. Except for the rigid boundary, all experimental conditions and equipment were kept the same as those used in the experiment of this study. The measurement results for the maximum bubble radius are shown in Figure 7. The range of the maximum bubble radius was 59.1–61.2 mm. The average value of the maximum radius of the bubble $R_{max}$ was 60 mm. Since the maximum error of $R_{max}$ was 1.2 mm (2%), this value was considered valid. Therefore, it is reasonable to assume that $R_{max}$ = 60 mm.

Based on the above algorithm, we obtained the corresponding relationship between the dimensionless parameters *γ* and the explosion distance *d,* as shown in Table 2. The relationship between the dimensionless parameters *ζ* and the boundary radius *r* is shown in Table 3. Here, the round flat board radius was considered to be infinite (∞).

### 2.6. “Pressure–Time” Curve Processing

**Figure 8:**
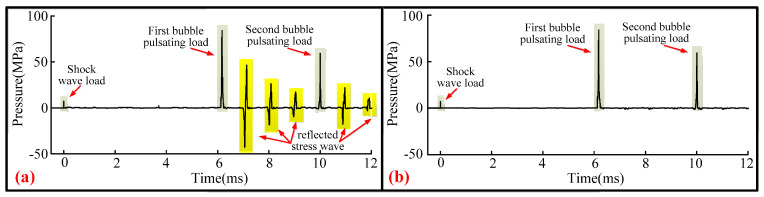
The pressure–time curve measured by the Hopkinson bar. (**a**) The pressure–time curve of the complete signal measured by the Hopkinson bar and (**b**) the optimized reflected stress wave signal, leaving only the pressure–time curve of the incident stress wave signal.

The pressure curve measured using HPB is shown in Figure 8a. This curve has both an initial stress wave and a reflected stress wave. This paper only studied the initial stress wave. Therefore, to highlight the shockwave load and bubble collapse load, Figure 8a was simplified into Figure 8b. In other words, the pressure–time curve measured by the HPB in this paper only shows the incident stress wave generated by an underwater explosion.

Here, the stress wave encountered structural discontinuities, such as cross sections, during the propagation process. Thus, the stress wave will undergo reflection and transmission [28,29,30,31]. These phenomena will have an impact on the bubble pulsation process, which is inevitable for the bubble pulsation process near the boundary. Young-Lin Yoo et al. [32] found that when a reflected shockwave with a large load propagates to the bubble wall, reflected and transmitted waves will be generated inside the bubble. BAI Li-xin et al. [33] found that the effect of reflected shockwaves on bubbles when the load is large can cause the formation of reverse bubble jets. However, the length of the HPB in this study was 2.5 m, which was thicker than many borders. Energy attenuation occurs when the stress wave propagates in the HPB. When the stress wave propagates to the end of the HPB, part of the energy will also be transmitted into the water and lost. When the stress wave is reflected from the HPB’s tail-end to the HPB’s measurement end surface, reflection and transmission will occur. The energy that will eventually be transmitted to the water and affect bubble pulsation will then be relatively small. Thus, in this experiment, the amplitude of the shockwave reflected into the water was low, with little diffusion and dissipation. Consequently, the influence on the bubbles was minimal and can be ignored.

## 3. Results and Discussion

The experimental results show that different *ζ* and *γ* values will affect the bubble dynamics and load characteristics. When both *ζ* and *γ* change, the movement mode of the bubbles will become very complicated. However, these modes can be broadly divided into four categories, as shown in Table 4. Each type has its own characteristics to be studied.

### 3.1. Effect of Different ζ Values on Bubble Dynamics

#### 3.1.1. The Case of *γ* = 0.33

**Figure 9:**
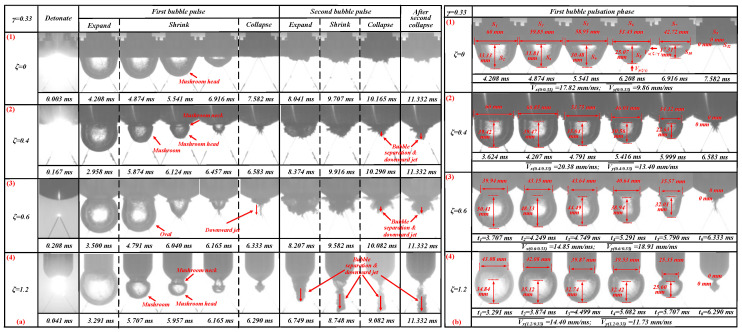
(**a**) The bubble’s morphological changes under the influence of different *ζ* values. The first row shows the bubble’s morphological changes when *ζ* = 0 and *γ* = 0.33. The second row shows the bubble’s morphological changes when *ζ* = 0.4 and *γ* = 0.33. The third row shows the bubble’s morphological changes when *ζ* = 0.6 and *γ* = 0.33. The fourth row shows the bubble’s morphological changes when *ζ* = 1.2 and *γ* = 0.33. (**b**) The bubble’s contraction speed under the influence of different *ζ* values. The first row shows the bubble’s contraction speed when *ζ* = 0 and *γ* = 0.33. The second row shows the bubble’s contraction speed when *ζ* = 0.4 and *γ* = 0.33. The third row shows the bubble’s contraction speed when *ζ* = 0.6 and *γ* = 0.33. The third row shows the bubble’s contraction speed when *ζ* = 1.2 and *γ* = 0.33.

The bubble dynamics of 0.33 ≤ *γ* ≤ 0.5 were basically the same. This section used *γ* = 0.33 to refer to this type. The bubble pulsation processes for *γ* = 0.33 with *ζ* = 0, 0.4, 0.6, and 1.2 are shown in Figure 9.

When *ζ* = 0 and *γ* = 0.33, before the bubble expanded to its maximum volume, the upper part of the bubble came into contact with the flat plate, as shown in Figure 9a (1). In the shrinking phase, the upper part of the bubble was always in contact with flat plate due to the proximity of the explosion’s location to the flat plate. When *t* = 4.874–6.961 ms, the bubbles show an inverted mushroom head shape. Judging from the shooting results of the high-speed camera, there was no obvious jetting phenomenon in the whole process. When *t* = 0.003 ms, the bubbles began to form, and when *t* = 7.582 ms, the bubbles collapsed for the first time. Therefore, the time of the first bubble pulsation process was 7.589 ms. The time of the bubble’s first pulsation period was represented by $T_{p\left(\zeta /\gamma \right)}$. $T_{p\left(0/0.33\right)}$ = 7.579 ms.

When *ζ* = 0.4 and *γ* = 0.33, the bubble expanded on the curved boundary surface (see Figure 9a (2)). In the shrinking phase, a mushroom head appeared at the bottom of the bubble, and a short mushroom neck appeared at the top of the bubble. Since the contact section between the bubble and the boundary surface shrank faster along the boundary surface than on other parts of the bubble, the upper part of the bubble formed a “neck” (see t = 5.874–6.457 ms). The shooting results of the high-speed camera indicate that the bubble did not produce an obvious jet during the first pulsation. During the second collapse, compared to *ζ* = 0, the downward bubble produced a small amount of bubble separation and a downward jet (see *t* = 10.290–11.332 ms). When *t* = 0.167 ms, bubbles began to form, and when *t* = 6.290 ms, the bubbles collapsed for the first time. Therefore, the time of the bubble’s first pulsation period was 6.416 ms ($T_{p\left(0.4/0.33\right)}$ = 6.416 ms), and when *ζ* = 0.4 and *γ* = 0.33, the time of the bubble’s first pulsation period was less than *ζ* = 0 *γ* = 0.33. That is, $T_{p\left(0.4/0.33\right)}$ < $T_{p\left(0/0.33\right)}$.

When *ζ* = 0.6 and *γ* = 0.33, the bubble also expanded on the boundary surface (see Figure 9a (3)). Unlike the first two cases, the bubble here exhibited an oval contraction during its first shrinking phase (see *t* = 4.791 ms) with a clear upward jet (see *t* = 6.165 ms; the upward jet velocity was 214.4 m/s, and the upward jet velocity algorithm will be introduced in Section 3.1.2.). During the starting stage of the jet, the bubbles showed an inverted cone shape (see *t* = 6.040 ms). At the formation stage of the jet, the bubbles assumed the shape of an inverted bell (at *t* = 6.165 ms). During the second collapse, compared with *ζ* = 0 and 0.4, the downward bubbles produced more obvious bubble separation and a downward jet (see *t* = 10.082–11.332 ms). When *t* = 0.208 ms, bubbles began to form, and when *t* = 6.333 ms, the bubbles collapsed for the first time. Therefore, the time of the bubble’s first pulsation period was 6.125 ms ($T_{p\left(0.6/0.33\right)}$ = 6.125 ms). Thus, when *ζ* = 0.6 and *γ* = 0.33, the time of the bubble’s first pulsation period was less than *ζ* = 0.4 and *γ* = 0.33. That is, $T_{p\left(0.6/0.33\right)}$ < $T_{p\left(0.4/0.33\right)}$ < $T_{p\left(0/0.33\right)}$.

When *ζ* = 1.2 and *γ* = 0.33, the bubble expanded to its maximum volume, and the upper part of the bubble completely wrapped around the bottom of the curved surface boundary (see Figure 9a (4)). In the shrinking phase, there were more obvious mushroom heads and mushroom necks compared to the *ζ* = 0 and 0.4 bubbles (see *t* = 5.707–6.165 ms). In the expansion stage of the second pulsation, there was obvious bubble separation and downward jetting (see *t* = 6.749 ms). In the subsequent pulsation process, the phenomena of bubble separation and downward jetting became more obvious (see *t* = 8.748–11.332 ms). Thus, when 0.33 ≤ *γ* ≤ 0.5, as *ζ* increased, the bubble separation and downward jet phenomena became increasingly more obvious. When *t* = 0.041 ms, bubbles began to form, and when *t* = 6.333 ms, the bubbles collapsed for the first time. Therefore, the time of the bubble’s first pulsation period was 6.249 ms ($T_{p\left(1.2/0.33\right)}$ = 6.249 ms). Although the time of the bubble’s first pulsation period when *ζ* = 1.2 and *γ* = 0.33 was slightly longer than that when *ζ* = 0.6 and *γ* = 0.33, it remained less than that when *ζ* = 0.4 and *γ* = 0.33. That is, $T_{p\left(1.2/0.33\right)}$ < $T_{p\left(0.4/0.33\right)}$ < $T_{p\left(0/0.33\right)}$. This shows that with an increase of *ζ*, the time of the bubble’s first pulsation period gradually decreased, which supports the perspective of Y. Tomita et al. [16]. That is, as the boundary curvature increases, the bubble pulsation time gradually decreases due to a decrease in the boundary inertia.

We maintained that the differences in the shrinkage shape of the bubble in the first stage might be caused by the shrinkage speeds of different parts of the bubble. Suppose that the average value of the shrinkage speed’s horizontal projection on the lateral bubble surface along the curved surface boundary (*x* direction) is $V_{x\left(\zeta /\gamma \right)}$. The average shrinkage speed of the bubble’s bottom point to the curved surface’s boundary bottom (y direction), then, is $V_{y\left(\zeta /\gamma \right)}$. The formulas for solving $V_{x\left(\zeta /\gamma \right)}$ and $V_{y\left(\zeta /\gamma \right)}$ are as follows:

(2) $$V_{x\left(\zeta /\gamma \right)}\;=\;\sum_{1}^{n}\left|\frac{\left(S_{2n+1}-S_{2n-1}\right)/\left(t_{n+1}-t_{n}\right)}{n}\right|$$

(3) $$V_{y\left(\zeta /\gamma \right)}\;=\;\sum_{1}^{n}\left|\frac{\left(S_{2n+2}-S_{2n}\right)/\left(t_{n+1}-t_{n}\right)}{n}\right|$$

where $S_{2n+1}$ represents the circular cross-section diameter of the curved surface boundary wrapped by the bubble’s upper part, and $S_{2n}$ represents the straight-line distance from the bottom of the curved surface boundary to the bottom of the bubble, as shown in Figure 9b (1). To further generalize the selected time $t_{n}$, the time should be divided among the bubble’s first shrinking phase equally.

Through measurements and calculations, it was found that when *ζ* = 0, in the first pulsation phase, $V_{x\left(0/0.33\right)}$ = 17.82 mm/ms and $V_{y\left(0/0.33\right)}$ = 9.86 mm/ms; when ζ = 0.4, in the first pulsation phase, $V_{x\left(0.4/0.33\right)}$ = 20.38 mm/ms and $V_{y\left(0.4/0.33\right)}$ = 13.40 mm/ms; when ζ = 0.6, in the first pulsation phase, $V_{x\left(0.6/0.33\right)}$ = 14.85 mm/ms and $V_{y\left(0.6/0.33\right)}$ = 18.91 mm/ms; and when ζ = 1.2, in the first pulsation phase, $V_{x\left(1.2/0.33\right)}$ = 14.40 mm/ms and $V_{y\left(1.2/0.33\right)}$ = 11.73 mm/ms. Thus, when $V_{x\left(\zeta /\gamma \right)}$ > $V_{y\left(\zeta /\gamma \right)}$, the bubbles were more likely to shrink into an “inverted mushroom shape” in the first pulsation phase. When $V_{x\left(\zeta /\gamma \right)}\;<\;V_{y\left(\zeta /\gamma \right)}$, the bubbles were more likely to shrink into an oval shape during the first pulsation phase.

**Figure 10:**
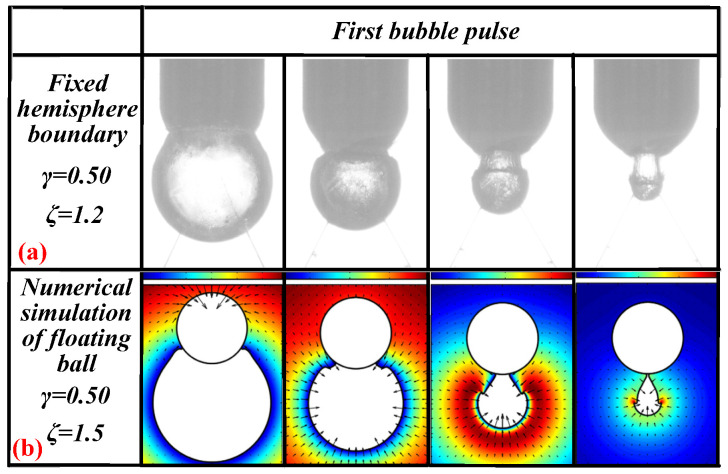
(**a**) The bubble’s morphological changes under the influence of *ζ* = 1.2 and *γ* = 0.50. (**b**) Numerical simulation of the bubble’s morphological changes. The pictures are quoted from [17].

Li Shuai et al. [17] used numerical methods to simulate the interactions between bubbles and suspended spheres. These numerical simulation results are shown in Figure 10b. Figure 10b features pictures quoted from [17]. The conditions of Li Shuai et al.’s numerical simulation are *γ* = 0.50 and *ζ* = 1.5, which represent the boundary of the suspended sphere. These values are similar to the *γ* = 0.50 and *ζ* = 1.2 values used in this study for the fixed hemisphere boundary. The bubble’s morphological changes in this experiment are shown in Figure 10a. Comparing the two showed that the morphological changes of the bubbles are almost identical. This proves that the numerical simulation used by Li Shuai et al. is correct.

#### 3.1.2. The Case of *γ* = 0.83

**Figure 11:**
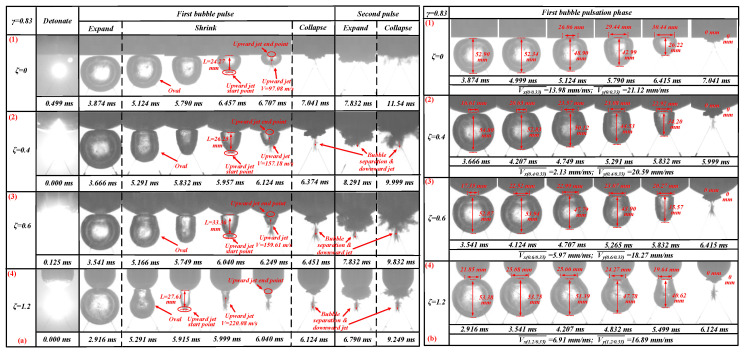
(**a**) The bubble’s morphological changes under the influence of different *ζ* values. The first row shows the bubble’s morphological changes when *ζ* = 0 and *γ* = 0.83. The second row shows the bubble’s morphological changes when *ζ* = 0.4 and *γ* = 0.83. The third row shows the bubble’s morphological changes when *ζ* = 0.6 and *γ* = 0.83. The fourth row shows the bubble’s morphological changes when *ζ* = 1.2 and *γ* = 0.83. (**b**) The bubble’s contraction speed under the influence of different *ζ* values. The first row shows the bubble’s contraction speed when *ζ* = 0 and *γ* = 0.83. The second row shows the bubble’s contraction speed when *ζ* = 0.4 and *γ* = 0.83. The third row shows the bubble’s contraction speed when *ζ* = 0.6 and *γ* = 0.83. The third row shows the bubble’s contraction speed when *ζ* = 1.2 and *γ* = 0.83.

The bubble dynamics and load characteristics of 0.67 ≤ *γ* ≤ 0.83 were basically the same. This section used *γ* = 0.83 to refer to this type. The bubbles’ pulsation process when *γ* = 0.83 with *ζ* = 0, 0.4, 0.6, and 1.2 is shown in Figure 11. 

Similar results to the shrinking process of the bubble’s first pulsation are observed in Figure 11a (1) (as in Figure 11a (3)). When *ζ* = 0 and *γ* = 0.83, the shrinking process of the first cycle of the bubble presents an oval shape. At the same time, there is an obvious jet phenomenon in the shrinking process during the bubble’s first pulsation at *t* = 6.457–6.707 ms. When *t* = 6.457 ms, the upward jet begins to form; the upward jet’s starting point is located at the bottom of the bubble, as shown in Figure 11a (1). At this stage, the distance *L* between the starting point of the upward jet and the bottom of the boundary was measured. Distance ‘*L*’ is the stroke of the upward jet passing through. When *t* = 6.707 ms, the upward jet ended, and the upward jet’s end point was located at the bottom of the boundary. The upward jet was formed at a time of 6.457 ms, and the upward jet ended at 6.707 ms, so the time ‘*t*’ from the beginning to the end of the upward jet cycle was 0.25 ms. The stroke of the upward jet was measured as 24.27 mm. Thus, the jet velocity generated can be obtained by $V_{uj\left(\zeta /\gamma \right)}\;=\;L/t$. That is, $V_{uj\left(0/0.83\right)}$ = 97.08 m/s. During the pulsation process of the first cycle and the second cycle of the bubble, no obvious downward jet was produced. The bubble shape in the upward jet stage was the same as that shown in Figure 9a (3).

With an increase in the ζ value, the bubble’s morphological changes shown in Figure 11a (2) were largely the same as those in Figure 11a (1) with some variations. When the bubble’s upward jet was formed, the bottom of the bubble was sharper than when *ζ* = 0 and *γ* = 0.83 at *t* = 5.957 ms. Compared to *ζ* = 0 and *γ* = 0.83, the bubble here experienced bubble separation and a downward jet during the first collapse and second pulsation stage at *t* = 6.374–9.999 ms. Using the same method, the upward jet velocity $V_{uj\left(0.4/0.83\right)}$ generated when *ζ* = 0.4 and *γ* = 0.83 was 157.18 m/s. $V_{uj\left(0.4/0.83\right)}\;>\;V_{uj\left(0/0.83\right)}$. The bubble shape in the upward jet stage was the same as that shown in Figure 11a (1).

Figure 11a (3) shows a similar contraction to that illustrated in Figure 11a (2). During the first contraction of the bubble, the bubble also presents an oval shape at *t* = 5.166 ms. Unlike *ζ* = 0.4 and *γ* = 0.83, here, when the bubble formed an upward jet in the first stage, the bottom of the bubble was sharper at *t* = 6.040 ms. The bubbles at this time (*t* = 6.040–6.249 ms) were thinner and longer than those when *ζ* = 0.4 and *γ* = 0.83 (*t* = 5.957–6.124 ms). Compared to *ζ* = 0.4 and *γ* = 0.83, the bubble separation and downward ejection were more obvious here at t = 6.451–9.832 ms. Using the same method, it can be seen that the upward jet velocity $V_{uj\left(0.6/0.83\right)}$ generated when *ζ* = 0.6 and *γ* = 0.83 was 159.61m/s. $V_{uj\left(0.6/0.83\right)}$*_)_* > $V_{uj\left(0.4/0.83\right)}$ > $V_{uj\left(0/0.83\right)}$. The bubble shape in the upward jet stage was the same as that shown in Figure 11a (2).

As the ζ value grows even larger (as in Figure 11a (4)), the bubble shrank into an oval shape again (see *t* = 5.291 ms). Compared to Figure 11a (3), when the bubble forms an upward jet, the bottom of the bubble will be sharper (see *t* = 5.915 ms). The bubbles at this time (*t* = 5.915–6.040 ms) were thinner than those when *ζ* = 0.6 and *γ* = 0.83 (*t* = 6.040–6.249 ms). Compared with *ζ* = 0.6 and *γ* = 0.83, the bubble separation and downward ejection here were more obvious (see *t* = 6.124–9.249 ms). Using the same method, it can be seen that the upward jet velocity $V_{uj\left(1.2/0.83\right)}$ generated when *ζ* = 1.2 and *γ* = 0.83 was 220.08 m/s. $V_{uj\left(1.2/0.83\right)}$ > $V_{uj\left(0.6/0.83\right)}$ > $V_{uj\left(0.4/0.83\right)}$ > $V_{uj\left(0/0.83\right)}$. As ζ increased, the jet velocity $V_{uj\left(\zeta /\gamma \right)}$ became increasingly larger. The bubble shape in the upward jet stage was the same as that shown in Figure 11a (3).

Through measurements and calculations, we found that when *ζ* = 0, in the first pulsation phase, $V_{x\left(0/0.83\right)}$ = 13.98 mm/ms and $V_{y\left(0/0.83\right)}$ = 21.12 mm/ms (see Figure 11b (1)); when *ζ* = 0.4, in the first pulsation phase, $V_{x\left(0.4/0.83\right)}$ = 2.13 mm/ms and $V_{y\left(0.4/0.83\right)}$ = 20.59 mm/ms (see Figure 11b (2)); when *ζ* = 0.6, in the first pulsation phase, $V_{x\left(0.6/0.83\right)}$ = 5.97 mm/ms and $V_{y\left(0.6/0.83\right)}$ = 18.27 mm/ms (see Figure 11b (3)); and when *ζ* = 1.2, in the first pulsation phase, $V_{x\left(1.2/0.83\right)}$ = 6.91 mm/ms and $V_{y\left(1.2/0.83\right)}$ = 16.89 mm/ms (see Figure 11b (4)). These four kinds of bubbles shrank into an ‘oval shape’ (see Figure 11a). This verifies the conclusion above: when $V_{x\left(\zeta /\gamma \right)}<V_{y\left(\zeta /\gamma \right)}$, the bubbles were more likely to shrink into an ‘oval shape’ during the first pulsation phase.

Thus, the effect of *ζ* and *γ* was clearly significant. Under the influence of *ζ*, the bubble shape, upward jet, and bubble contraction velocity along the *x* direction and *y* direction, as well as the bubble separation and downward jet phenomena, all have regular changes. However, these situations all happen when the explosion source was very close to the boundary—that is, when the top of the bubble was in contact with the boundary in the first pulsation stage. Subsequent experiments were inspired by this phenomenon and were focused on determining the situation where the top of the bubble did not touch the boundary during the first pulsation stage. In this case, we found that the bubble shape, upward jet, and bubble contraction velocity along the *x* direction and *y* direction, as well as the bubble separation and downward jet phenomenon, experienced the same regular changes as those in the above experiment.

#### 3.1.3. The Case of *γ* = 1.33

**Figure 12:**
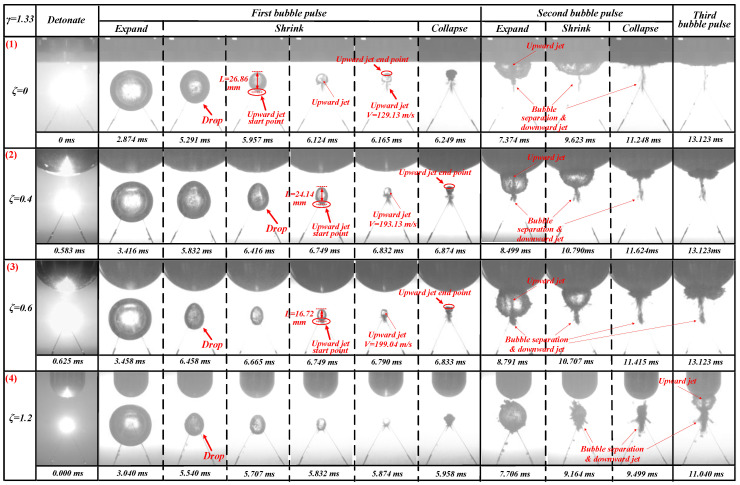
Bubble’s morphological changes under the influence of different *ζ* values. The first row shows the bubble’s morphological changes when *ζ* = 0 and *γ* = 1.33. The second row shows the bubble’s morphological changes when *ζ* = 0.4 and *γ* = 1.33. The third row shows the bubble’s morphological changes when *ζ* = 0.6 and *γ* = 1.33. The fourth row shows the bubble’s morphological changes when *ζ* = 1.2 and *γ* = 1.33.

The bubble dynamics and load characteristics of 1.00 ≤ *γ* ≤ 1.50 were basically the same. This section used *γ* = 1.33 to refer to this type. The bubbles’ pulsation process when *γ* = 1.33 with *ζ* = 0, 0.4, 0.6, and 1.2 is shown in Figure 12.

The contraction shape of the bubble in the first pulsation stage in Figure 12 (1) was different from that in Figure 9a and Figure 11a. The bubble in Figure 12 (1) shrank like a drop in the first pulsation stage at *t* = 5.291 ms. The upward jet can be seen during the shrinking phase of the first cycle of the bubble at *t* = 6.124 ms. At *t* = 5.957 ms, one can observe the beginning of the upward jet at the bottom of the bubble. At *t* = 6.165 ms, the upward jet hit the top of the bubble. In the pulsation phase of the bubble second cycle, an obvious upward jet was visible at *t* = 7.374 ms. At the same time, there was also a weak bubble separation and downward jet phenomenon during this stage at *t* = 7.374–11.248 ms. Based on our calculations, the upward jet velocity $V_{uj\;\left(0/1.33\right)}$ generated by the bubble in the shrinking phase of the first pulsation was 129.13 m/s.

When *ζ* = 0.4 and *γ* = 1.33, the contraction shape of the bubble in the first stage was more like a drop shape than that when *ζ* = 0 and *γ* = 1.33. Here, the top of the bubble was sharper, and the bottom was fuller at *t* = 6.416 ms. When *t* = 6.832 ms (Figure 12 (2)), the upward jet can also be seen, but the image clarity was weaker than that when *ζ* = 0 and *γ* = 1.33. At *t* = 6.749 ms, one can see the beginnings of the upward jet at the bottom of the bubble. At *t* = 6.874 ms, the upward jet hit the top of the bubble. When *t* = 8.499 ms, obvious upward jet, bubble separation, and downward jet phenomenon can be observed, which were clearer here than at *ζ* = 0 and *γ* = 1.33. When *t* = 10.790–11.624 ms, bubble separation and the downward jet phenomenon occurred, which were also clearer than those at *ζ* = 0 and *γ* = 1.33. Based on our calculations, the upward jet velocity $V_{uj\;\left(0.4/1.33\right)}$ generated by the bubble in the shrinking phase of the first pulsation was 193.13 m/s. $V_{uj\;\left(0.4/1.33\right)}$ > $V_{uj\;\left(0/1.33\right)}$.

The bubble shrinkage shape, jets, and other phenomena shown in Figure 12 (3) were very similar to those in Figure 12 (2). However, they were also different. First, the upward jet generated during the first pulsation and contraction of the bubble was less obvious. Secondly, the upward jet, bubble separation, and downward jet phenomena generated during the bubble’s second pulsation were also more obvious. Finally, $V_{uj\;\left(0.6/1.33\right)}$ = 199.04 m/s > $V_{uj\left(0.4/1.33\right)}$ > $V_{uj\left(0/1.33\right)}$ as ζ increased, the jet velocity $V_{uj\left(\zeta /\gamma \right)}$ became increasingly larger.

The contraction shape of the bubble in Figure 12 (4) at the first pulsation contraction stage was the same as that in Figure 12 (1)–(3) but with more differences. Here, no upward jet can be seen during the shrinking stage of the bubble’s first pulsation at *t* = 5.540–5.874 ms, and no upward jet can be seen during the second pulse of the bubble at *t* = 7.706 ms. Here, the bubble separation and downward jet phenomena were more obvious at *t* = 9.164–9.499 ms. In the bubble’s second pulsation stage, the top of the bubble can never touch the bottom of the boundary. However, after the bubble’s second pulsation stage, the bubble will produce upward jet, bubble separation, and downward jet phenomena, which are different from those in Figure 12 (1)–(3) at *t* = 11.040 ms. We hypothesized that this occurs due to the Bjerknes force.

After a series of experiments, we obtained the following change law. As with *γ* = 0.83, with an increase of *ζ*, the upward jet velocity generated by the bubble in the first pulsation stage will increase (not including the case of *ζ* = 1.2). With an increase of *ζ*, the bubble separation and downward jet phenomena produced by the bubble in the second pulsation stage will become increasingly more obvious. Unlike *γ* = 0.83, the bubble will shrink in the form of a drop during the first pulsation stage, which is a relatively novel phenomenon. With an increase of *γ*, several differences appeared in the properties of the bubbles. We also found that the Bjerknes force will also somewhat affect the formation of jets. To further verify our conjecture, we continued to increase the *γ* value for the following experiments.

#### 3.1.4. For the Case of *γ* = 2.00

**Figure 13:**
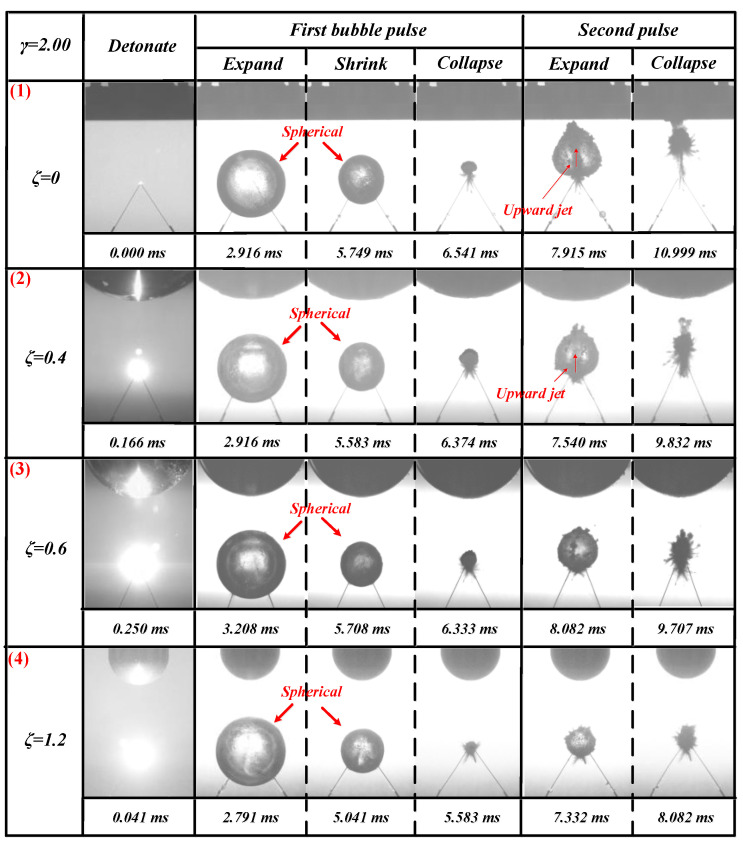
Bubble morphological changes under the influence of different *ζ* values. The first row shows the bubble’s morphological changes when *ζ* = 0 and *γ* = 2.00. The second row shows the bubble’s morphological changes when *ζ* = 0.4 and *γ* = 2.00. The third row shows the bubble’s morphological changes when *ζ* = 0.6 and *γ* = 2.00. The fourth row shows the bubble’s morphological changes when *ζ* = 1.2 and *γ* = 2.00.

The bubble dynamics and load characteristics of 1.67 ≤ *γ* ≤ 2.00 were basically the same. This section used *γ* = 2.00 to refer to this type. The bubbles’ pulsation process when *γ* = 2.00, with *ζ* = 0, 0.4, 0.6, and 1.2 is shown in Figure 13.

Figure 13 (1) shows that, in the detonation stage, the intersection of the copper tubes emitted a strong light and started to form bubbles. During the expansion phase of the first cycle of bubbles, the bubble expanded in a spherical shape. After the bubble expanded to its maximum volume, the bubble shrank into a spherical shape. No significant jets were generated at this stage. In the expansion stage of the bubble’s second pulsation, an obvious upward jet was produced at *t* = 7.915 ms.

The observations in Figure 13 (2) and Figure 13 (1) were very similar to those in the first pulsation period of the bubble. However, in Figure 13 (2), during the expansion stage of the bubble’s second pulsation, the upward jet produced was obviously weaker than that in Figure 13 (1) at *t* = 7.540 ms.

The data in Figure 13 (3) and (4) and Figure 13 (1) and (2) were also very similar to the results for the first pulsation period of the bubble. However, the results were completely different in the second stage. Figure 13 (3) and (4) did not produce an upward jet in the expansion stage of the bubble’s second pulsation (see Figure 13 (3) *t* = 8.082 ms and Figure 13 (4) *t* = 7.332 ms).

Since *γ* is the same, the explosion source of each bubble was also the same; only *ζ* was different. The collapse position of the first pulse of each bubble was also the same (at the intersection of the discharge needle). By summarizing the experimental results, we found that the distance of the upward jet caused by the second pulsation process of the bubble caused by the Bjerknes force was 1.67 ≤ *γ* ≤ 2.00, and the curvature was 0 ≤ *ζ* ≤ 0.4. That is, when 1.67 ≤ *γ* ≤ 2.00, the smaller *ζ* is, the easier it becomes to generate upward jets during the bubble’s second pulsation.

#### 3.1.5. Typical Bubble Shape

**Figure 14:**
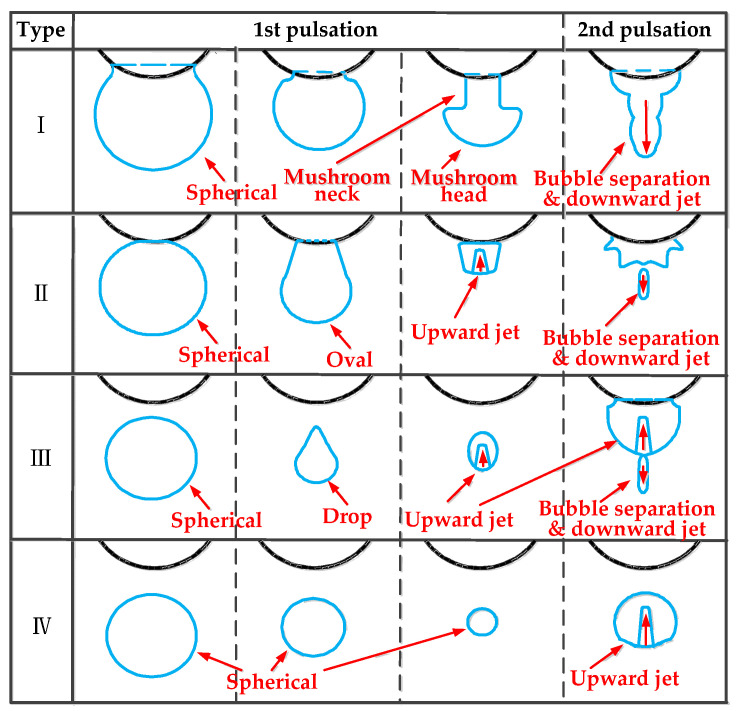
Bubble morphological change descriptions for types I–IV.

Through an analysis of the above experiments, we found that bubble dynamics not only depend on *γ* but also on *ζ*. This conclusion is the same as that of Y. Tomita et al. [16]. By summarizing the results of the 44 groups of experiments, we found that although the ζ and γ of each group of experiments are different, some of the experimental results have similarities. These experimental results can be divided into the following four categories, as shown in Figure 14. Type I refers to an “inverted mushroom shape” bubble. When 0 ≤ *ζ* ≤ 0.4 and 0.33 ≤ *γ* ≤ 0.50, *ζ* = 1.2, and 0.33 ≤ *γ* ≤ 0.50, a bubble will appear. Due to the close distance between the bubble and the boundary surface, the bubble will expand in a spherical shape and wrap around the bottom of the boundary during the expansion process of the first bubble pulsation. During the shrinking process of the first bubble pulsation, an obvious “inverted mushroom shape” will be produced, and an obvious “mushroom head” and “mushroom neck” will be seen. During the second bubble pulsation, obvious bubble separation and downward jet phenomena will sometimes occur. Type II refers to an oval-shaped bubble. This bubble will appear when 0 ≤ *ζ* ≤ 1.2 and 0.67 ≤ *γ* ≤ 0.83, *ζ* = 0.6, and 0.33 ≤ *γ* ≤ 0.50. The bubble will expand in a spherical shape during the first pulsation, but because the *γ* value in this case is larger than that of Type I, the top of the bubble can only wrap around a small part of the bottom of the boundary. Sometimes the bubble top is tangential to the boundary bottom and will shrink into an oval shape during the first pulsation phase of the bubble. At the same time, an obvious upward jet formation process will be observable. During the second bubble pulsation, there will be obvious bubble separation and the downward jet phenomenon will occur. Type III refers to a drop-shaped bubble. When 0 ≤ *ζ* ≤ 1.2 and 1.00 ≤ *γ* ≤ 1.50, bubbles will appear. In the first pulsation stage of the bubble, the bubble will expand into a spherical shape and contract into a drop shaped. When the bubble expands into a spherical shape, it will not touch the bottom of the boundary. During the contraction process, an upward jet will appear, albeit less clearly than that of Type II. In the second pulsation stages of the bubble, an upward jet and downward jet will appear at the same time. This phenomenon is very unique. Type IV refers to a spherical bubble. When 0 ≤ *ζ* ≤ 1.2 and 1.67 ≤ *γ* ≤ 2.00, these bubbles will appear. Due to the large explosion distance, the Bjerknes force here has little effect on the bubble, so this type of bubble will expand and shrink into a spherical shape during the first pulsation phase. In the second pulsation phase, an upward jet will appear.

#### 3.1.6. Time of the Bubble’s First Pulse Process

**Figure 15:**
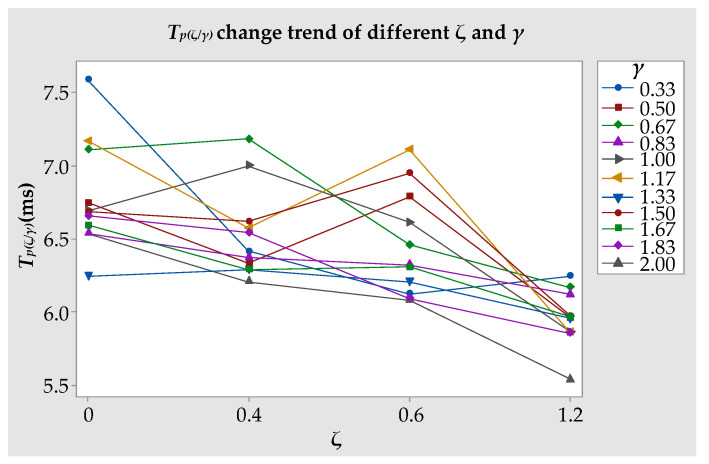
$T_{p\left(\zeta /\gamma \right)}$ change trends under different *ζ* and *γ* values.

As stated in Section 3.1.1. The time of the bubble’s first pulsation period is represented by $T_{p\left(\zeta /\gamma \right)}$. We summarized the times of the bubbles’ first pulsation periods over the 44 experiments and synthesized them into change trends, as shown in Figure 15 (see Appendix C for all data). From Figure 15, we can see that when *γ* was the same, as *ζ* increased, the overall trend of the curve decreased. However, there were individual points that slightly increased with an increase of *ζ*. For example, $T_{p\left(0.6/0.33\right)}$ = 6.125 ms, and $T_{p\left(1.2/0.33\right)}$ = 6.249 ms. By analyzing these data, the data at the turning point were shown to be slightly larger than the previous data. These turning points will not affect the overall change trends. Thus, as *ζ* increased, the bubble’s first pulsation time gradually decreased due to a decrease in the boundary inertia.

Best and Blake [34] derived the dimensionless period of the first pulsation of a bubble in 1994 as

(4) $$T_{p\left(\zeta /\gamma \right)}\;=\;\frac{2}{\sqrt{6}}\left\{B\left(\frac{5}{6},\frac{1}{2}\right)+\frac{1}{2}\mu B\left(\frac{7}{6},\frac{1}{2}\right)\right\}\;=\;1.829\left(1+0.4065\mu \right)$$

where *B* represents the incomplete function beta. The leading part on the right side of Equation (4) has the same period as that of the Rayleigh bubble [35]. Moreover, μ represents the extension factor. The solving equation for the μ of the sphere is as follows:

(5) $$\mu \;=\;\frac{a^{*}}{\gamma \left(2a^{*}+\gamma \right)}-\frac{1}{a^{*}}\ln\left\{\frac{{\left(a^{*}+\gamma \right)}^{2}}{\gamma \left(2a^{*}+\gamma \right)}\right\}$$

where $a^{*}\;=\;r/R_{max}\;=\;1/\zeta$. Thus, Equation (5) can be derived into the following equation:

(6) $$\mu \;=\;\frac{a^{*}}{\gamma \left(2a^{*}+\gamma \right)}-\frac{1}{a^{*}}\ln\left\{\frac{{\left(a^{*}+\gamma \right)}^{2}}{\gamma \left(2a^{*}+\gamma \right)}\right\}$$

Next, we can bring Equation (6) into Equation (4):

(7) $$T_{p\left(\zeta /\gamma \right)}\;=\;1.829\left\{1+0.4065\left[\frac{1}{\gamma \left(2+\zeta \gamma \right)}-\zeta \ln\left(\frac{{\left(1+\zeta \gamma \right)}^{2}}{\zeta \gamma \left(2+\zeta \gamma \right)}\right)\right]\right\}$$

Equation (7) is only suitable for calculating the bubble period near the boundary of the sphere, so the value range of *ζ* was 0.4, 0.6, and 1.2, while the value range of *γ* in this study was 0.33, 0.50, 0.67, 0.83, 1.00, 1.17, 1.33, 1.50, 1.67, 1.83, and 2.00. Next, we put *ζ* and *γ* into Equation (7) to calculate the value ($T_{p\left(\zeta /\gamma \right)}^{*}$) for the time of the bubble’s first pulsation period near the spherical surface. Equation (7) calculation results are shown in Table 5.

We could then compare the experimental measurement value with the calculated value of the equation and multiply Equation (7) by the correction coefficient to obtain the following equation:

(8) $$T_{p\left(\zeta /\gamma \right)}\;=\;1.199\left\{1+0.4065\left[\frac{1}{\gamma \left(2+\zeta \gamma \right)}-\zeta \ln\left(\frac{{\left(1+\zeta \gamma \right)}^{2}}{\zeta \gamma \left(2+\zeta \gamma \right)}\right)\right]\right\}+5.2$$

**Figure 16:**
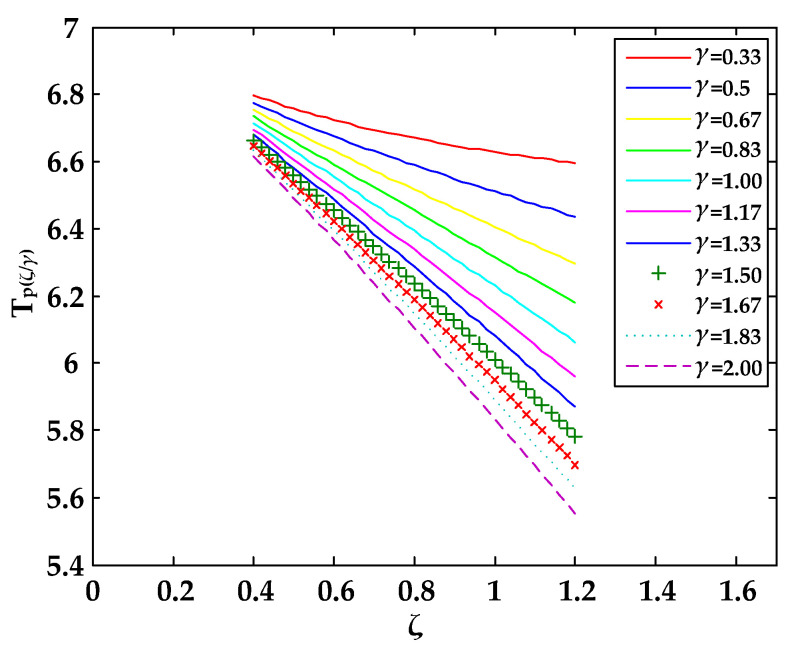
Fitting curve of the periodic equation of the first pulse of the bubble.

The value range of *γ* in this study was 0.33, 0.50, 0.67, 0.83, 1.00, 1.17, 1.33, 1.50, 1.67, 1.83, and 2.00. Thus, we substituted these values into Equation (8) and drew them onto a curve together to obtain Figure 16.

By comparing the fitted curve (Figure 16) with the actual curve (Figure 15), it was found that although there was still a slight error in the numerical value, the trend of the curve was the same. Whether Equation (8) can predict the first pulsation period of the bubbles near the hemisphere boundary of different curvatures needs to be tested by increasing the amount of experiments. This goal is worthy of a future study.

### 3.2. Effects of Different ζ and γ Values on the Load Characteristics

**Figure 17:**
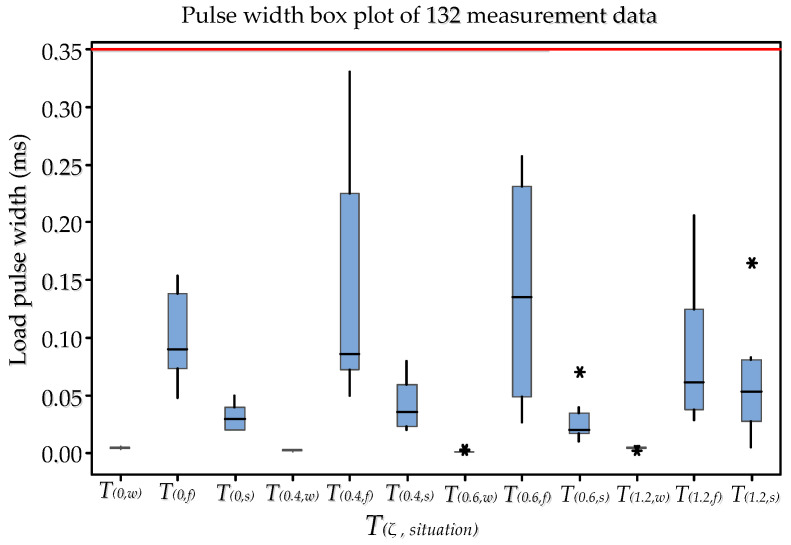
The pulse width box plot of 132 measurement data. The ordinate in this figure represents the load pulse width, with the units in ms. The abscissa in the figure represents $T_{\left(\zeta ,\;\mathrm{situation}\right)}$ and ζ represents curvature. “Situation” is represented by “w”, “f”, and “s”, where “w” is the pulse width of the shockwave load, “f” is the pulse width of the first bubble collapse load, and “s” is the pulse width of the second bubble collapse load. “*” represents the outlier in the box plot.

According to the pressure curve measured by the HPB, we measured the pulse width of the shockwave load, as well as the pulse widths of the first and second collapse loads of the bubble. Here, the range of pulse width was 0.001 ms ≤ *T* ≤ 0.330 ms. The specific situation is shown in Figure 17 and Appendix B. According to the conclusions in Section 2.2, when the load pulse width was less than $T_{HPB}$ (0.96 ms), the measured data were valid. Therefore, the 132 sets of pressure load data measured in this study were valid.

**Figure 18:**
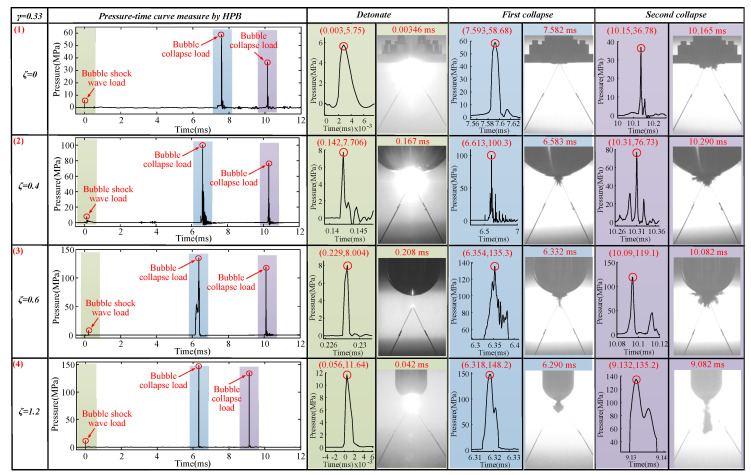
Pressure–time diagram for *γ* = 0.33 and different *ζ* values. The first row shows the pressure–time curves for the states of *ζ* = 0 and *γ* = 0.33. The second row shows the pressure–time curves for the states of *ζ* = 0.4 and *γ* = 0.33. The third row shows the pressure–time curves for the states of *ζ* = 0.6 and *γ* = 0.33. The fourth row shows the pressure–time curves for the states of *ζ* = 1.2 and *γ* = 0.33.

The shockwave load and bubble collapse load measured by HPB when *γ* = 0.33 are shown in Figure 18. The load pulse widths measured by the HPB (0.001 ms ≤ *T* ≤ 0.258 ms) were less than $T_{HPB}$ (0.96 ms), as shown in Figure 18. Therefore, the pressure load measured by the HPB was valid. When *ζ* = 0, the shockwave load, first bubble collapse load, and second bubble collapse load were 5.75, 58.68, and 36.78 MPa, respectively, as shown in Figure 18 (1). When *ζ* = 0.4, the loads measured in the three stages were 7.706, 100.3, and 76.73 MPa, respectively, as shown in Figure 18 (2). When *ζ* = 0.6, the loads measured in the three stages were 8.004, 135.3, and 119.1 MPa, respectively, as shown in Figure 18 (3). When *ζ* = 1.2, the loads measured in the three stages were 11.64, 148.2, and 135.2 MPa, respectively, as shown in Figure 18 (4). With an increase of *ζ*, the shockwave load, the first bubble collapse load, and the second bubble collapse load show an increasing trend. Notably, there was an obvious bimodal phenomenon in the first bubble collapse stage at *ζ* = 0.6, which might be related to the upward jet flow generated by the bubble at this time. Thus, both the bubble collapse load and the upward jet load were measured by the HPB.

**Figure 19:**
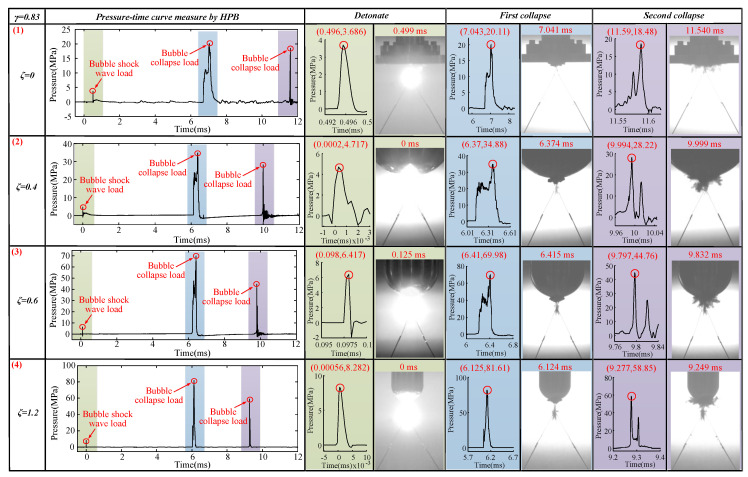
Pressure–time diagram for *γ* = 0.83 and different *ζ* values. The first row shows the pressure–time curves for the states of *ζ* = 0 and *γ* = 0.83. The second row shows the pressure–time curves for the states of *ζ* = 0.4 and *γ* = 0.83. The third row shows the pressure–time curves for the states of *ζ* = 0.6 and *γ* = 0.83. The fourth row shows the pressure–time curves for the states of *ζ* = 1.2 and *γ* = 0.83.

**Figure 20:**
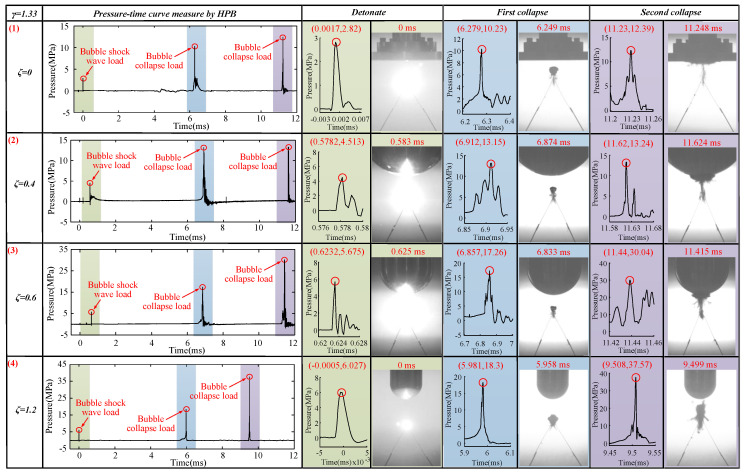
Pressure–time diagram for *γ* = 1.33 and different *ζ* values. The first row shows the pressure–time curves for the states of *ζ* = 0 and *γ* = 1.33. The second row shows the pressure–time curves for the states of *ζ* = 0.4 and *γ* = 1.33. The third row shows the pressure–time curves for the states of *ζ* = 0.6 and *γ* = 1.33. The fourth row shows the pressure–time curves for the states of *ζ* = 1.2 and *γ* = 1.33.

**Figure 21:**
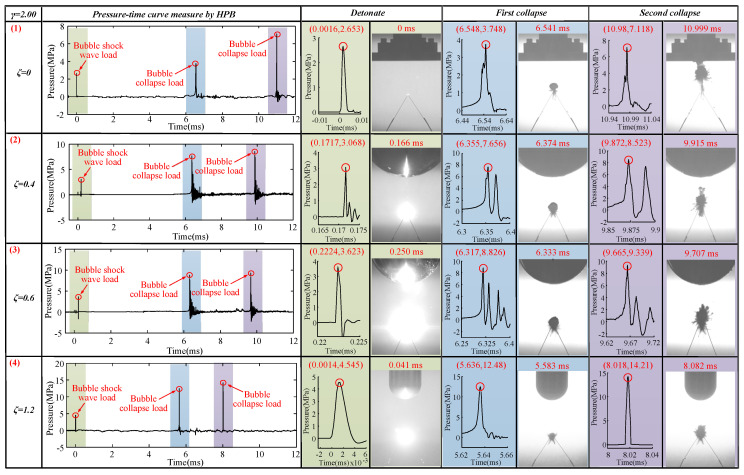
Pressure–time diagram for *γ* = 2.00 and different *ζ* values. The first row shows the pressure–time curves for the states of *ζ* = 0 and *γ* = 2.00. The second row shows the pressure–time curves for the states of *ζ* = 0.4 and *γ* = 2.00. The third row shows the pressure–time curves for the states of *ζ* = 0.6 and *γ* = 2.00. The fourth row shows the pressure–time curves for the states of *ζ* = 1.2 and *γ* = 2.00.

To further verify our conclusions, we continued to use HPB to measure different combinations of *ζ* and *γ*. The results are shown in Figure 19, Figure 20 and Figure 21. The load pulse widths measured by the HPB (0.001 ms ≤ *T* ≤ 0.330 ms) were less than $T_{HPB}$ (0.96 ms), as shown in Figure 19, Figure 20 and Figure 21. Therefore, the load measured by the HPB was valid. Thus, based on the measurement results, whether *γ* = 0.83, 1.33, or 2.00, the pressure load change law was the same as the results obtained above. That is, as *ζ* increased, the shockwave load, first bubble collapse load, and second bubble collapse load all show an increasing trend. At the same time, when *γ* = 0.83, due to the upward jets of bubbles near various boundaries at this time, there were obvious double peaks in the bubble pressure–time curve, as shown in Figure 19. When *γ* = 1.33, with an increase of *ζ*, the upward jet phenomenon gradually disappeared along with a gradual disappearance of the multipeak phenomena of the first collapse of the bubble, as shown in Figure 20. Based on this experimental result, we posit that an obvious upward jet will cause the bubble double peak phenomenon.

**Figure 22:**
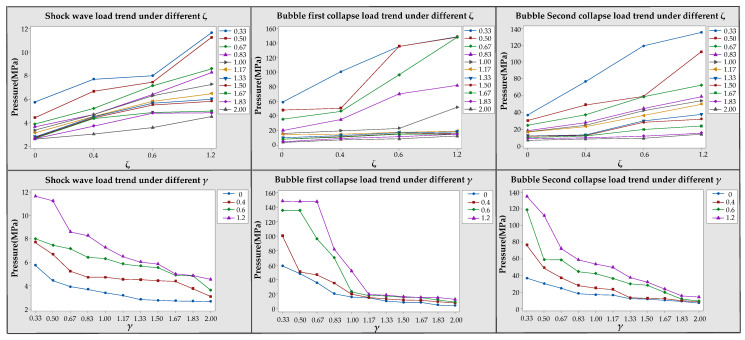
Load trend for 0.33 ≤ *γ* ≤ 2.00 and 0 ≤ *ζ* ≤ 1.2.

The shockwave load, first bubble collapse load, and second bubble collapse load curves in the 44 experiments are plotted in Figure 22. As can be seen from Figure 22, with the increase of *γ*, the overall shockwave load, first bubble collapse load, and second bubble collapse load gradually decreased. In the experiment, some second bubble collapse loads were bigger than the first bubble collapse loads with an increase of *γ*. This is because the bubble moved upward after the first pulsation, causing the second collapse position to be closer to the measurement end-face of the HPB. With an increase of *ζ*, the shockwave load and bubble collapse load will increase. The pressure load data measured by the HPB when 0 ≤ *ζ* ≤ 1.2 and 0.33 ≤ *γ* ≤ 2.00 are provided in Appendix A.

The results of the jet velocity in Section 3.1.2 and Section 3.1.3 were next combined with the pressure load measurement results for a comprehensive analysis. We found that the faster the jet velocity is, the greater the pressure load it generates, which is consistent with the conclusions of Tomita et al. [16]. At the same time, we also observed the effect of curvature on the jet velocity and bubble pressure load. As ζ increased, the jet velocity and bubble pressure load became increasingly larger.

According to the above experimental results, several bubble load characteristics were found: (1) When *γ* was the same, with an increase of *ζ*, the shockwave load, the first bubble collapse load, and the second bubble collapse load show an upward trend. (2) If there is an obvious upward jet, the pressure–time curve of the bubble measured by the HPB is likely to have double peaks. (3) When *ζ* was the same, as *γ* increased, the shockwave load and the peaks of the first and second pulsation loads of the bubble gradually decreased.

## 4. Conclusions

In this study, we used high-speed cameras and HPB measurement technology to conduct a series of experimental studies on the bubble dynamics and load characteristics of electric spark bubbles under different curvature boundaries and different explosion distances. The following important conclusions were drawn based on a statistical analysis of the experimental results:

(1) Under the same premise of *γ*, with an increase of *ζ*, the shockwave load and bubble collapse load will increase. Under the same premise of *ζ*, with an increase of *γ*, the shockwave load and the bubble collapse loads gradually decrease. If there is an obvious upward jet, the pressure–time curve of the bubble measured by HPB is likely to have double peaks.

(2) The bubble shape will change according to changes in the bubble shrinkage speed and *γ*. When $V_{x\left(\zeta /\gamma \right)}>V_{y\left(\zeta /\gamma \right)}$, the bubbles are more likely to shrink into an inverted mushroom shape. When $V_{x\left(\zeta /\gamma \right)}<V_{y\left(\zeta /\gamma \right)}$, the bubbles are more likely to shrink into an oval shape. When 1.00 ≤ *γ* ≤ 1.50, the bubbles are more likely to shrink in a drop shaped. When 1.67 ≤ *γ* ≤ 2.00, the bubbles are more likely to shrink into a spherical shape. Under the same premise of *γ*, with an increase of ζ, the time of the bubble’s first pulsation period gradually decreases due to a decrease of the boundary inertia.

(3) When oval-type and drop-type bubbles appear, they are the most likely to produce a clear upward jet during the first pulse period of the bubble. In the first pulse period of the bubble, under the same premise of *γ*, with an increase of *ζ*, the upward jet velocity will increase. In the second pulse period of the bubble, under the same premise of *γ*, the upward jet will gradually weaken with an increase of *ζ*, while the downward jet and bubble separation phenomena will become increasingly more obvious.

In this paper, the effects of different *ζ* and *γ* values on the bubble dynamics and load characteristics were studied experimentally, and, through the experimental results, we summarized the change laws of some physical quantities. In future research, we will use mathematical models to simulate the experimental results. However, whether a mathematical model is feasible and whether the simulation results can meet the relevant requirements will require specific experiments to verify. Moreover, whether Equation (8) can predict the time of the bubble’s first pulsation period near the hemisphere boundaries of different curvatures needs to be tested in a future study by increasing the amount of experiments. We hope that our research will contribute to the antiriot design of ship bottoms.

## Figures and Tables

**Table 1:** The properties of aluminum, steel, and copper.

Name	Modulus of Elastic (GPa)	Thin Rod Longitudinal Wave Velocity (km/s)
aluminum	70.3	5.1
copper	129.8	3.8
steel	211.0	5.2

**Table 2:** Correspondence of *γ* and *d*.

*γ*	*d* (mm)	*γ*	*d* (mm)	*γ*	*d* (mm)	*γ*	*d* (mm)
0.33	10	0.83	25	1.33	40	1.83	55
0.50	15	1.00	30	1.50	45	2.00	60
0.67	20	1.17	35	1.67	50		

**Table 3:** Correspondence of *ζ* and *r*.

*ζ*	*r* (mm)	*ζ*	*r* (mm)	*ζ*	*r* (mm)	*ζ*	*r* (mm)
0	∞	0.4	25	0.6	50	1.2	75

**Table 4:** Experimental cases.

No.	*ζ*	*γ*	Type		No.	*ζ*	*γ*	Type	
1	0	0.33	I	Inverted mushroom bubble	23	0.6	0.33	II	Oval bubble
2		0.50			24		0.50		
3		0.67	II	Oval bubble	25		0.67		
4		0.83			26		0.83		
5		1.00	III	Drop-shaped bubble	27		1.00	III	Drop-shaped bubble
6		1.17			28		1.17		
7		1.33			29		1.33		
8		1.50			30		1.50		
9		1.67	IV	Spherical bubble	31		1.67	IV	Spherical bubble
10		1.83			32		1.83		
11		2.00			33		2.00		
12	0.4	0.33	I	Inverted mushroom bubble	34	1.2	0.33	I	Inverted mushroom bubble
13		0.50			35		0.50		
14		0.67	II	Oval bubble	36		0.67	II	Oval bubble
15		0.83			37		0.83		
16		1.00	III	Drop-shaped bubble	38		1.00	III	Drop-shaped bubble
17		1.17			39		1.17		
18		1.33			40		1.33		
19		1.50			41		1.50		
20		1.67	IV	Spherical bubble	42		1.67	IV	Spherical bubble
21		1.83			43		1.83		
22		2.00			44		2.00		

**Table 5:** Equation (7) calculation results.

No.	*γ*	*ζ*	$T_{p\left(\zeta /\gamma \right)}^{*}(\mathrm{ms})$	*ζ*	$T_{p\left(\zeta /\gamma \right)}^{*}(\mathrm{ms})$	*ζ*	$T_{p\left(\zeta /\gamma \right)}^{*}(\mathrm{ms})$
1	0.33	0.4	2.435	0.6	2.076	1.2	2.127
2	0.50		2.152		1.974		1.959
3	0.67		2.029		1.924		1.897
4	0.83		1.927		1.894		1.870
5	1.00		1.901		1.875		1.855
6	1.17		1.885		1.864		1.846
7	1.33		1.872		1.855		1.841
8	1.50		1.863		1.849		1.838
9	1.67		1.857		1.845		1.835
10	1.83		1.852		1.842		1.834
11	2.00		2.322		2.076		1.833

## Appendix A

**Table A1:** Wall pressure loads under different *ζ* and *γ* values.

No.	*ζ*	*γ*	Load (MPa)			No.	*ζ*	*γ*	Load (MPa)		
			Wave	First	Second				Wave	First	Second
1	0	0.33	5.750	58.68	36.78	23	0.6	0.33	8.004	135.3	119.1
2		0.50	4.443	48.11	30.45	24		0.50	7.450	135.2	59.02
3		0.67	3.907	35.35	24.64	25		0.67	7.156	96.15	58.70
4		0.83	3.686	20.11	18.48	26		0.83	6.417	69.98	44.76
5		1.00	3.384	15.86	17.02	27		1.00	6.302	22.94	42.28
6		1.17	3.172	14.55	16.67	28		1.17	5.857	17.93	36.52
7		1.33	2.820	10.23	12.39	29		1.33	5.675	17.26	30.04
8		1.50	2.750	8.300	11.79	30		1.50	5.537	15.52	28.29
9		1.67	2.705	8.210	10.26	31		1.67	4.899	14.93	19.90
10		1.83	2.675	4.760	9.074	32		1.83	4.838	11.72	11.90
11		2.00	2.653	3.750	7.118	33		2.00	3.623	8.826	9.339
12	0.4	0.33	7.706	100.3	76.73	34	1.2	0.33	11.64	148.2	135.2
13		0.50	6.673	50.51	49.20	35		0.50	11.23	147.9	112.1
14		0.67	5.226	46.28	37.15	36		0.67	8.588	147.6	72.39
15		0.83	4.717	34.88	28.22	37		0.83	8.282	81.61	58.85
16		1.00	4.710	19.72	24.88	38		1.00	7.265	51.74	53.80
17		1.17	4.541	14.91	23.33	39		1.17	6.485	19.25	50.00
18		1.33	4.513	13.15	13.24	40		1.33	6.027	18.30	37.57
19		1.50	4.429	11.57	12.78	41		1.50	5.860	16.30	32.23
20		1.67	4.387	11.05	12.36	42		1.67	5.008	15.00	23.80
21		1.83	3.751	9.234	9.890	43		1.83	4.859	14.73	15.47
22		2.00	3.068	7.656	8.523	44		2.00	4.545	12.48	14.21

## Appendix B

**Table A2:** Load pulse widths under different *ζ* and *γ* values.

No.	*ζ*	*γ*	Load Pulse Width (ms)			No.	*ζ*	*γ*	Load Pulse Width (ms)		
			Wave	First	Second				Wave	First	Second
1	0	0.33	0.005	0.091	0.030	23	0.6	0.33	0.001	0.258	0.010
2		0.50	0.005	0.138	0.040	24		0.50	0.001	0.160	0.030
3		0.67	0.005	0.154	0.020	25		0.67	0.001	0.243	0.010
4		0.83	0.005	0.146	0.020	26		0.83	0.001	0.231	0.017
5		1.00	0.006	0.090	0.030	27		1.00	0.001	0.145	0.020
6		1.17	0.005	0.076	0.030	28		1.17	0.001	0.135	0.020
7		1.33	0.004	0.106	0.030	29		1.33	0.001	0.069	0.020
8		1.50	0.005	0.048	0.050	30		1.50	0.001	0.054	0.070
9		1.67	0.004	0.085	0.020	31		1.67	0.002	0.049	0.040
10		1.83	0.005	0.073	0.040	32		1.83	0.003	0.028	0.027
11		2.00	0.003	0.070	0.040	33		2.00	0.001	0.027	0.035
12	0.4	0.33	0.002	0.081	0.060	34	1.2	0.33	0.004	0.047	0.081
13		0.50	0.003	0.086	0.030	35		0.50	0.004	0.062	0.025
14		0.67	0.002	0.318	0.040	36		0.67	0.005	0.125	0.083
15		0.83	0.002	0.330	0.023	37		0.83	0.005	0.206	0.055
16		1.00	0.002	0.225	0.070	38		1.00	0.006	0.160	0.047
17		1.17	0.002	0.112	0.040	39		1.17	0.004	0.077	0.164
18		1.33	0.002	0.092	0.020	40		1.33	0.004	0.080	0.053
19		1.50	0.001	0.068	0.080	41		1.50	0.002	0.043	0.081
20		1.67	0.003	0.072	0.030	42		1.67	0.003	0.029	0.028
21		1.83	0.003	0.075	0.020	43		1.83	0.004	0.038	0.054
22		2.00	0.001	0.050	0.036	44		2.00	0.005	0.030	0.005

## Appendix C

**Table A3:** The existence time of the first bubble pulse.

No.	*γ*	*ζ*	$T_{p\left(\zeta /\gamma \right)}(\mathrm{ms})$	*ζ*	$T_{p\left(\zeta /\gamma \right)}(\mathrm{ms})$	*ζ*	$T_{p\left(\zeta /\gamma \right)}(\mathrm{ms})$	*ζ*	$T_{p\left(\zeta /\gamma \right)}(\mathrm{ms})$
1	0.33	0	7.579	0.4	6.416	0.6	6.125	1.2	6.249
2	0.50		6.749		6.334		6.790		5.963
3	0.67		7.111		7.183		6.461		6.171
4	0.83		6.542		6.374		6.326		6.124
5	1.00		6.694		7.002		6.614		5.868
6	1.17		7.172		6.576		7.109		5.865
7	1.33		6.249		6.291		6.208		5.958
8	1.50		6.685		6.622		6.950		5.975
9	1.67		6.596		6.291		6.309		5.969
10	1.83		6.659		6.545		6.096		5.857
11	2.00		6.541		6.208		6.083		5.542
